# Nanodelivery systems: An efficient and target‐specific approach for drug‐resistant cancers

**DOI:** 10.1002/cam4.6502

**Published:** 2023-09-05

**Authors:** Sumel Ashique, Ashish Garg, Afzal Hussain, Arshad Farid, Prashant Kumar, Farzad Taghizadeh‐Hesary

**Affiliations:** ^1^ Department of Pharmaceutics Pandaveswar School of Pharmacy Pandaveswar India; ^2^ Guru Ramdas Khalsa Institute of Science and Technology, Pharmacy Jabalpur India; ^3^ Department of Pharmaceutics, College of Pharmacy King Saud University Riyadh Saudi Arabia; ^4^ Gomal Center of Biochemistry and Biotechnology Gomal University Dera Ismail Khan Pakistan; ^5^ Teerthanker Mahaveer College of Pharmacy Teerthanker Mahaveer University Moradabad India; ^6^ Department of Pharmaceutics, Amity Institute of Pharmacy Amity University Madhya Pradesh (AUMP) Gwalior India; ^7^ ENT and Head and Neck Research Center and Department, The Five Senses Health Institute, School of Medicine Iran University of Medical Sciences Tehran Iran; ^8^ Clinical Oncology Department Iran University of Medical Sciences Tehran Iran

**Keywords:** cancer, chemotherapy, drug delivery, drug resistance, nanomedicine

## Abstract

**Background:**

Cancer treatment is still a global health challenge. Nowadays, chemotherapy is widely applied for treating cancer and reducing its burden. However, its application might be in accordance with various adverse effects by exposing the healthy tissues and multidrug resistance (MDR), leading to disease relapse or metastasis. In addition, due to tumor heterogeneity and the varied pharmacokinetic features of prescribed drugs, combination therapy has only shown modestly improved results in MDR malignancies. Nanotechnology has been explored as a potential tool for cancer treatment, due to the efficiency of nanoparticles to function as a vehicle for drug delivery.

**Methods:**

With this viewpoint, functionalized nanosystems have been investigated as a potential strategy to overcome drug resistance.

**Results:**

This approach aims to improve the efficacy of anticancer medicines while decreasing their associated side effects through a range of mechanisms, such as bypassing drug efflux, controlling drug release, and disrupting metabolism. This review discusses the MDR mechanisms contributing to therapeutic failure, the most cutting‐edge approaches used in nanomedicine to create and assess nanocarriers, and designed nanomedicine to counteract MDR with emphasis on recent developments, their potential, and limitations.

**Conclusions:**

Studies have shown that nanoparticle‐mediated drug delivery confers distinct benefits over traditional pharmaceuticals, including improved biocompatibility, stability, permeability, retention effect, and targeting capabilities.

## 1 INTRODUCTION

Cancer accounts for a significant portion of worldwide deaths, expected to cause 17 million deaths by 2030.^1^ Despite recent advances, chemotherapy is still the mainstay of cancer treatment. However, it is often insufficiently selective for tumor cells resulting in various adverse effects like bone marrow suppression, neurointestinal and gastrointestinal toxicities, immunosuppression, alopecia, healing defect, and vomiting. Cancer cells may become resistant to various chemotherapeutics, developing multidrug resistance (MDR) and eventually leading to treatment failure.^2^ Drug resistance can develop after or even during therapy. Research has indicated that drug resistance accounts for over 90% of cancer patient deaths.^3^, ^4^ Numerous mechanisms execute this phenomenon, including improving DNA damage repair, blocking apoptotic cascade, altering drug target molecules, detoxifying drugs by various overexpressed proteins, and upregulation of adenosine triphosphate (ATP)‐binding cassette (ABC) pumps to efflux chemotherapeutics.^2^, ^5^ Low pH, abnormal vasculature, and localized hypoxia are additional changes in the tumor microenvironment (TME) impeding drug penetration.^6^, ^7^ To date, various strategies have been examined to avoid MDR, including small‐molecule inhibitors, chemosensitizers, and using gene therapy. Nevertheless, these approaches might harm patient lives at effective doses. In addition, combinational regimens can be used to concurrently target multiple signaling pathways, including chemodrugs in addition to tyrosine kinase inhibitors, P‐glycoprotein (P‐gp) inhibitors, and proapoptotic agents to increase cytotoxicity.^8^, ^9^ Nevertheless, due to the different pharmacokinetics and the required doses, the results of these treatments might be unpredictable.

The advances in nanotechnology have offered clinicians novel tools for fighting against MDR malignancies by providing direct drug accessibility to the tumor cells for an extended time, using immature leaky vessels and irregular lymphatic drainage of solid tumors to favor drug release.^10^, ^11^, ^12^ Numerous nanomaterials have been investigated to this end, ranging from inorganic (carbon nanotubes,^13^, ^14^ iron oxide nanomaterials,^15^ gold nanoparticulate systems,^16^, ^17^ complex quantum dots,^18^ and mesoporous silica‐based agents^19^, ^20^) to the organic nanoparticles (NPs) (liposomes,^21^, ^22^ polymer‐based NPs,^23^ micelles,^24^ and dendrimers^25^). However, nanocarriers that function solely as passive drug delivery systems pose significant limitations impeding their widespread use in oncology.^26^ NPs can discharge their contents during circulation or before approaching target tissues. This limitation can be solved using suitable targeting ligands for specific binding to cancer cells.^27^, ^28^ Drug endocytosis is made possible by active targeting to avoid drug efflux from cellular cytoplasm.^29^ Additionally, it has been possible to create stimuli‐responsive nanomedicines that can perform active drug transport and prompt the release of the drug once it reaches the targeted tumor.^30^, ^31^ During past decade, several researches have shown the effectiveness of NPs in overcoming MDR in different malignancies.^32^, ^33^ The most notable preclinical research studies are given, together with evidence of any potential limitations and highlights of the opportunities for future clinical applications in this review. Additionally, NPs have demonstrated some benefits in defeating MDR by providing a platform for combination drug therapy by preventing efflux transporters (present on the cell surface) mediated MDR.^34^ NP‐based treatment has been applied to defeat MDR in a multitude of cancer types like breast,^35^ ovarian,^36^ and prostate cancers.^37^ The use of nano‐therapeutics has enabled a new phase of cancer treatment, and the interdisciplinary nature of these two areas calls for additional research. This review explores diverse forms of inorganic as well as organic NPs and the most cutting‐edge strategies used to combat MDR as one of the most challenging defense mechanisms of cancer cells.^38^

## 2 MECHANISTIC PERSPECTIVE TO TARGET CANCER CELLS

**FIGURE 1:**
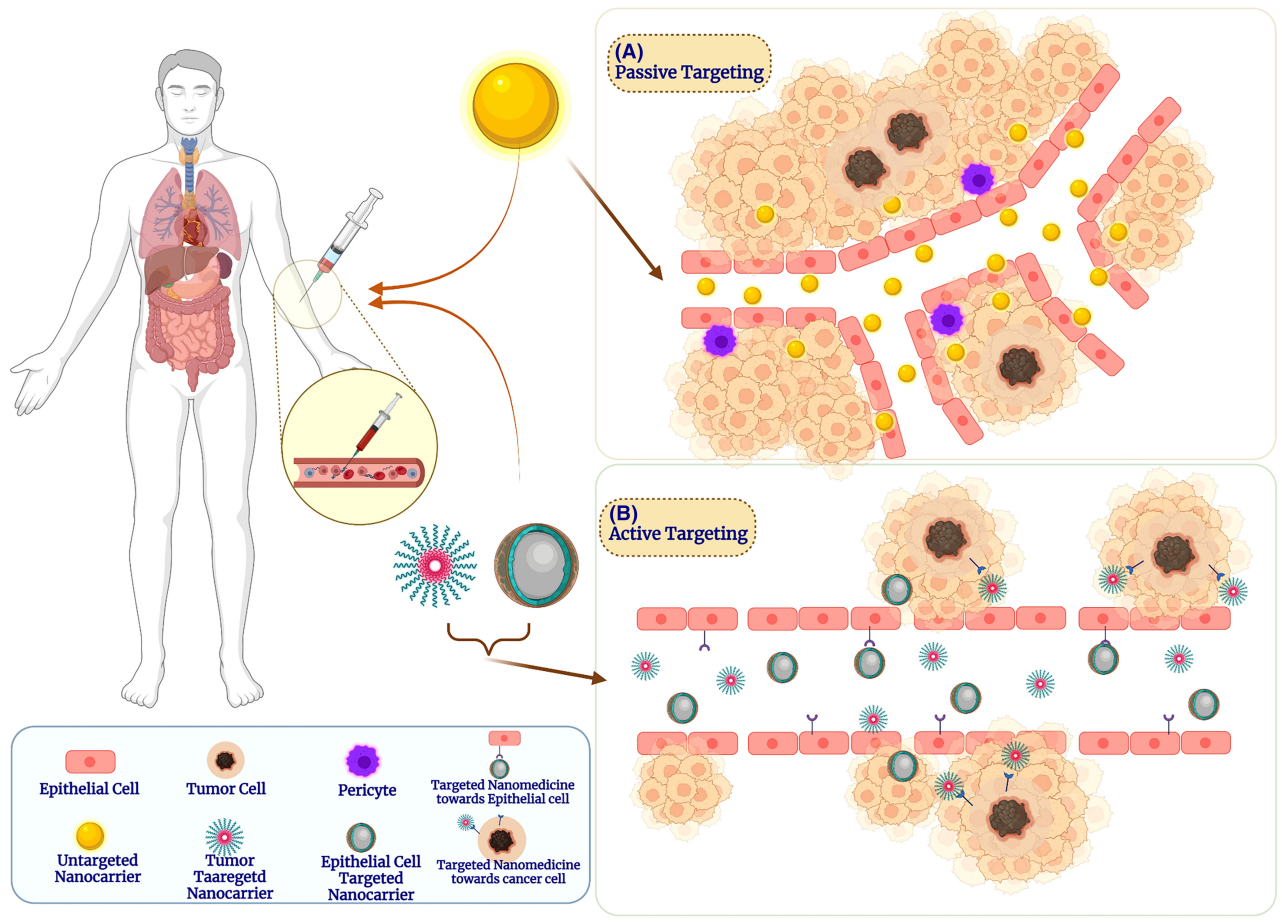
**(**A) Nanomedicine's theoretical passive targeting (EPR effect) is depicted schematically. (B) Nanomedicine that is actively targeted and grafted with a peptide or antibody that can bind to a particular receptor that is overexpressed by either cancer cells or endothelial cells.

A crucial aspect of nanocarriers for drug delivery is their ability to find and kill cancer cells, thereby enhancing therapeutic efficacy while safeguarding healthy cells from harm. Numerous investigations into the targeting architecture of NP‐based medicines have been conducted. Understanding the biology of tumors and how nanocarriers interact with cancer cells is crucial to tackle the difficulties of therapeutic applications and develop effective and efficient nanocarrier systems. Two broad categories of targeting mechanisms are categorized as passive targeting and active targeting **(**Figure 1 **)**:

### 2.1 Passive targeting

Passive targeting aims to exploit the differences between tumor and normal tissue. In this process, desired drug or drug‐loaded nanomedicine could be accessed through various modes without bioenergy expenses such as concentration gradients, pH differences (between physiological blood and TME), and enhanced penetration and retention (EPR) effect. Notably, large gaps between the endothelial cells of blood vessels with rapid cell proliferation promote neovascularization to worsen the selectivity of tumor arteries relative to healthy vessels, which develop undesirable side effects after chemotherapy.^39^ NPs can leak from these gaps to penetrate the tumor tissues and accumulate within TME for a prolonged time due to poor lymphatic drainage for sustained and delayed drug delivery. This process is called the EPR effect, which plays a key role in passive targeting.^40^ The NP size and morphology impact the EPR, required to be considered as determining factors in designing appropriate drug delivery systems through passive delivery. Generally, small NPs have superior permeability over larger NPs across these gaps of blood vessels. Therefore, NPs do not drain into healthy vasculature, favoring fewer side effects.^41^, ^42^ In addition, the functional immune system is more likely to get rid of bigger particles.^43^

Glycolysis is a prime energy source for tumor cells for growth, cell proliferation, angiogenesis, and metastasis.^44^ TME is crucial to the passive diffusion of nanomedicines per the EPR effect due to the lowered pH of the TME after glycolysis. Thus, the unique physiological acidic nature of TME can be used as a strategy for site‐specific drug delivery. Several studies implemented this concept in designing pH‐sensitive NPs for passive and active drug delivery.^45^ The acidosis of surrounding TME is an opportunity to administer pH‐responsive NPs that will release drugs specifically in acidic environments to improve drug access and reduce side effects. It has been demonstrated that TME (pH = 6.5–6.8) has the potential to facilitate precise and effective tumor imaging and therapy through various mechanisms by amplifying imaging signals, increasing NPs accumulation, and enabling deeper intratumoral penetration.^46^, ^47^

Hence, nanotechnology has made substantial progress using diverse nanomaterials, smart materials, and biomaterials to develop effective and efficient drug delivery, culminating in recent disease diagnosis and treatment challenges. These nanomaterials exhibit remarkable responsiveness to acidic pH conditions within tumors, as evidenced by their physicochemical attributes comprising surface charge and size and their ability to undergo acid‐triggered cleavage of covalent bonds and decomposition.^30^

### 2.2 Active targeting

The interactions involving ligands and receptors in active targeting allow for the selective targeting of cancer cells. The chemicals that highly attach to the surface of cancer cells are the receptors targeted NPs to distinguish between the target sites and healthy cells.^48^, ^49^ The interaction of NPs ligands and tumor cell receptors results in the internalization (endocytosis) of NPs and selective release of the therapeutic drugs into the target cells.^50^ Proteins, DNA, and small interfering RNAs (siRNAs) are macromolecular drugs, and active targeting for these is ideally suited for their intracellular delivery. Common examples of targeting structural moieties are monoclonal antibodies, polypeptides, glycoproteins, folate, and carbohydrates.^51^ These ligands strongly attach to the receptors on target cells and facilitate the internalization of NPs.

## 3 TARGETING CANCER‐SPECIFIC BIOMOLECULES

Transferrin is a specific class of serum glycoprotein responsible for carrying iron into cells. Most solid tumors have overexpressed transferrin receptors (about 40% of total cancer cells), whereas normal cells express them sparingly. As a result, transferrin‐conjugated NPs can enhance the selectivity of antitumor agents.^52^, ^53^ A demonstration of transferrin‐modified NPs has been made to have improved intracellular drug delivery and greater cellular absorption efficiency than non‐modified NPs.^54^ Besides, transferrin‐conjugated polymeric NPs can be administered to overcome chemotherapy resistance.^55^ Folic acid is taken up through specific receptor‐mediated absorption (only expressed in a few normal cells) to produce nucleotides in cancerous cells. It has been reported that α‐folate receptors (an isoform) are overexpressed on cancer cells (~40% of human cancers).^56^ Therefore, folate‐conjugated nanomaterials have been successfully employed to target the folate receptor for cancer treatment.^57^, ^58^ Similarly, various forms of glycoproteins are overexpressed on the surface, such as lectins, which are common non‐immunological complex proteins for identification.^59^ In contrast, the reverse lectin targeting mechanism targets the lectins present on sites of cancer cells with carbohydrate moieties integrated into NPs.^60^

The overexpressed epidermal growth factor receptor (EGFR) gene participates in a variety of tumor progression and development pathways. It has already been used as a candidate for cancer therapy.^61^ For instance, therapies targeting the human epidermal receptor 2 (HER2) are typically effective in HER2‐positive mammary and gastric cancers.^62^, ^63^ Therefore, a promising approach for medication delivery targets cancer cells that overexpress the EGFR receptor by using customized ligands that adhere to EGFR in NPs. It has been demonstrated that applying a combination of two cancer‐specific ligands into a single NPs can increase the target specificity.^64^

## 4 NANOCARRIERS TO OVERCOME MDR IN TUMOR MICROENVIRONMENT

This section provides a comprehensive summary of the available inorganic, organic, and hybrid nanomaterials to overcome MDR in cancer cells. The most notable preclinical research studies are provided, together with evidence of any potential limitations and highlights of the opportunities for future clinical applications. The failure of chemotherapy is often attributed to MDR, which negatively impacts the treatment outcomes. The mechanistic phenomenon of MDR can be explained as (a) activated efflux pumps to expel intracellular drug molecules from cells and (b) arrested apoptosis, reinforced DNA repair, and improved oxidation resistance. Nanocarriers have offered various benefits to overcome MDR through drug targeting, decreased efflux, and reduced drug toxicity and side effects. Chen et al. established a summary of the biological processes underlying tumor MDR and nano‐based treatment approaches aimed at reversing MDR. The authors concluded that nanomedicine can serve as a basis for advancing more efficient approaches to combat cancer.^65^

### 4.1 Inorganic nanocarriers

**FIGURE 2:**
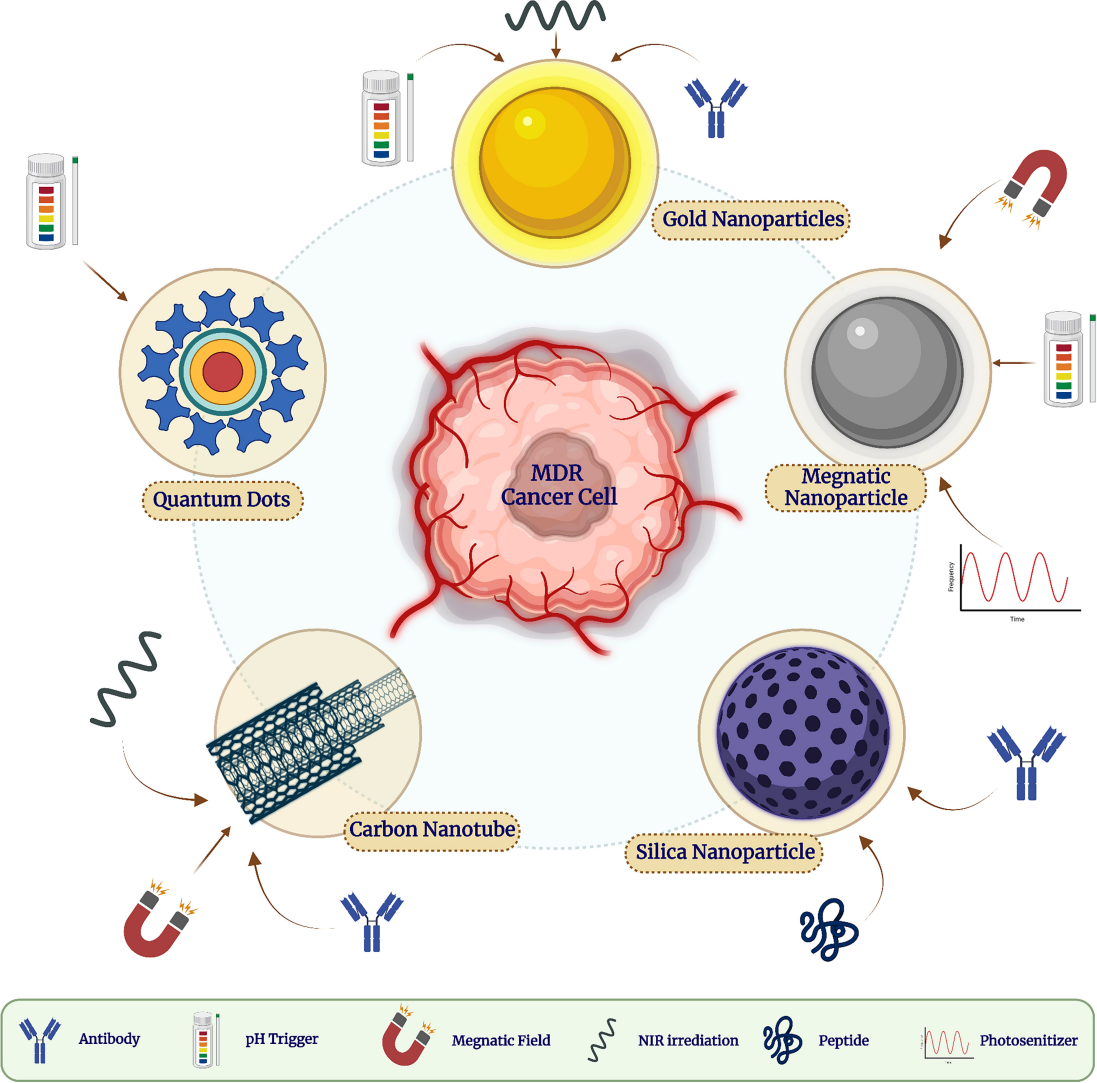
An illustration of the primary inorganic nanoparticles under investigation for battling MDR in cancer. Nanomedicines are shown in the diagram from left to right. There have been reports of potential functionalization using targeted ligands and external stimuli used to stimulate drug release. IO, iron oxide; MDR, multidrug resistance; NIR, near‐infrared.

Inorganic NPs can offer various benefits over traditional anticancer methods, for example, high surface‐to‐volume ratio, easy surface modification for in vivo MDR tumor treatment using particular ligands, and flexible characteristics responding to external stimuli, including temperature, magnetic field, and near‐infrared (NIR) light. The subgroups of inorganic NPs are as follows (Figure 2):

#### 4.1.1 Iron oxide and other metal oxide nanoparticles

Because of high biocompatibility, iron oxide NPs are frequently used in the biomedical industry. Superparamagnetic iron oxide nanoparticles (SPIONs) are metallic NPs applied as contrast agents, biosensors, and hyperthermia therapies.^66^ Additionally, metallic NPs were established as effective drug delivery systems by releasing their cargo primarily upon endocytosis, avoiding the complex pump‐based transporters found in MDR cancer cells. For instance, iron oxide NPs can enhance the doxorubicin uptake by chemoresistant HeLa cells, which results in apoptosis induction.^67^ Moreover, a pH‐sensitive typical hydrazone connection on the iron oxide NPs surface was exploited in an intriguing investigation to covalently bond doxorubicin to polyethyleneimine (PEI). Furthermore, endosomal escape and cumulative drug release in the cytoplasm of glioma cells from resistant rats have been seen after NPs based treatment, demonstrating greater cellular retention than free doxorubicin.^68^ Researchers have found an approach to use pH‐sensitive NPs using magnetic ingredients prone to disassemble in an acidic TME as imaging techniques for diagnosis and, after being loaded with photosensitizers that generate singlet oxygen, utilized in photothermal therapy to target cancerous cells.^69^ The use of combination therapy consisting of inhibitors and chemotherapeutics confronts a variety of challenges to overcome MDR by modulating various pharmacokinetic features of these classes of drugs.^70^ Notably, nanocarriers are well suited for this task as they may carry numerous molecules simultaneously and deliver them to targeted tissue.^71^ In a ground‐breaking study, 5‐bromo tetrandrine‐carrying iron oxide NPs effectively enhanced cytotoxic effects in MDR cell lines. It promoted daunorubicin accumulation and downregulated the overall expression of the P‐gp gene and the *mdr1* gene, aiding in the reversal of MDR.^72^ Another study found that cells readily incorporated Wogonin and daunorubicin after a co‐loading approach on iron oxide NPs, localized them in endosomes, triggered a considerable volume of apoptosis to reduce the leukemic cell line MDR1 mRNA transcription and P‐gp expression.^73^ The application of pyrophoric magnetic NPs in an alternating magnetic field to induce cancer cell apoptosis or hyperthermia is an effective approach to treating MDR tumors, particularly in stimulating the release of drugs.^74^ The ability to transport daunorubicin and 5‐bromo tetrandrine has been developed in iron oxide magnetic NPs. In an in vivo leukemia model, these agents were delivered in the context of an alternating magnetic field. Interestingly, MDR was reversed as the P‐gp and Bcl‐2 (B‐cell lymphoma 2) expression declined on the one hand, and caspase‐3 and Bax (Bcl‐2‐associated X protein) expression enhanced on the other hand.^75^ More recently, doxorubicin‐carrying NPs of iron oxide in silk fibroin exhibited cytotoxicity and intracellular accumulation when used on MDR breast cancer cells.^76^ Additionally, siRNAs have been successfully delivered by iron oxide NPs to decrease the expression of Bcl‐2, an antiapoptotic protein, to overcome MDR.^77^ Overall, these findings show that iron oxide NPs can effectively combat MDR. However, more structural development would be required to find the optimum carrier for an adequate dose. Additionally, it must be remembered that the external magnetic field used for hyperthermia treatments needs to be precisely adjusted regarding frequency and exposure time based on the specific nanosystems.^78^ The ability to reverse MDR has been demonstrated in other metal oxide‐based NPs. As an illustration, zinc oxide and copper oxide NPs were found to behave as chemosensitizers in vivo, impeding MDR transport processes.^79^ Polyethylene glycol (PEG)‐modified MnO_2_ nanosheets have been developed to quickly release doxorubicin in MDR cells. Due to this alteration, the nanomaterial was substantially brittle in an acidic intracellular environment and stable under physiological settings. Nanomaterials and their size‐mediated P‐gp efflux pump inhibition facilitate drug accumulation inside the cells, and Mn^2+^ ion aids sensitivity to MDR cancer cells.^80^ Guo et al. (2019) designed a nanocarrier by adjoining the iron–phenolic network with tannic acid for doxorubicin delivery in dendrimer. The construct was labeled as “Den–DOX–tannic acid–Fe^3+^” (DDTF) nanocomposite to treat MDR cancer cells.^81^ Doxorubicin treatment induces apoptosis in cancer cells due to a significant elevation in reactive oxygen species (ROS) levels. This elevated ROS level sensitizes cancer cells to ferroptosis via the Fenton reaction, ultimately enhancing the efficacy of chemodynamic therapy (CDT).^81^

#### 4.1.2 Gold nanoparticles

Gold NPs have been applied to produce stable and highly biocompatible nanocarriers with decent penetrability inside living cells.^82^, ^83^ A variety of payloads can be carried by tumor cells using this type of nanomaterial.^84^ Gold‐doxorubicin nanoconjugates have been developed for effective drug internalization and drug release medicines in MDR cells.^85^ MDR breast cancer cells can endocytose gold NP‐doxorubicin complex through an acid‐labile connector. Then, doxorubicin is efficiently released in acidic TME, leading to the apoptosis of target cells.^86^ In photothermal treatment (PTT), customized gold NPs can specifically kill cancer cells by converting absorbed near‐infrared light to heat.^87^ Its effective use in the preclinical setting has been documented.^88^ Anti‐death receptor‐4 (DR4) monoclonal antibodies functionalized with doxorubicin‐loaded gold NPs have been applied in a xenograft model to deliver a large drug dose to the tumor tissue using PTT phenomenon.^89^ Surface functionalization may cause NPs to become unstable. Biocompatible ingredients (polyethylene glycol and phospholipids) are essential for creating gold NPs with improved stability.^90^

#### 4.1.3 Quantum dots

Quantum dots (QDs) can be used for therapeutic and diagnostic purposes. Graphene quantum dots have recently been shown to effectively reverse the resistance to doxorubicin in selected MCF‐7/ADR cells via interrelating with the C‐rich sites of associated promoters to reduce the activity of P‐gp and MRP1.^91^ In A549 cells, P‐gp expression was significantly decreased by CdSe and ZnS‐MPA combining to CdSe/ZnS‐GSH quantum dots, while miR‐34b and miR‐185 were effectively increased. These findings demonstrate the potential of QDs to be involved in future cancer treatments.^92^ QDs have interesting optical and chemical properties for drug‐delivery systems that can obstruct MDR pathways by surface modification.^93^, ^94^ A novel study on HeLa cells applied a specific type of QDs (CdSe/ZnSe) to facilitate the delivery of doxorubicin and P‐gp‐targeting siRNAs. The investigators found that CdSe/ZnSe QDs could effectively reverse the MDR and provide real‐time tracking of the cancer cells.^95^ Even though the results are encouraging and new, research is still necessary to fully assess any potential adverse effects on the generation of ROS, the release of metal ions within cells, and the long‐term toxicity of QDs in living beings.^96^

#### 4.1.4 Mesoporous silica NPs

Different studies have explored the peculiar properties of mesoporous silica NPs (MSNs), such as the capacity to be functionalized for targeted administration, large pore volume and surface area that facilitate high drug loading, and a pore shape that may be customized.^97^ MSNs can facilitate the internalization of various biomolecules (monoclonal antibodies, peptides, and nucleic acids) through macropinocytosis.^98^ Due to the ability of MSNs to transport the carriers inside cells while shielding them from aberrant molecular pathways of MDR cancer cells, they have been frequently exploited for loading drugs intended for MDR reversal mediated via passive or active targeting modes.^99^ Doxorubicin was further incorporated into large pore MSNs and rapidly released at high concentrations, triggering P‐gp downregulation and MDR reversal.^100^ Rod‐shaped doxorubicin‐carrying MSNs were successfully absorbed by MDR breast carcinoma cells, whereas the co‐delivery of tetrandrine and paclitaxel ultimately overcame MDR in breast cancer cells and had significant therapeutic consequences.^101^, ^102^ A DNA damage repair inhibitor (arsenic trioxide, ATO) received FDA‐approval for acute promyelocytic leukemia. However, its results in solid tumors were not desirable due to low bioavailability and significant side effects. An experimental study showed that silica NPs simultaneously carrying doxorubicin and ATO can successfully eliminate the MDR hepatocellular carcinoma cells.^103^ In a study on MDR breast cancer cells, nuclear‐targeted doxorubicin‐loaded MSNs significantly enhanced cellular apoptosis.^104^ DNA repair pathways were changed due to MDR‐related gene expression, disrupting the tumor suppressor cascade dependent on the p53 base.^105^ Additionally, MSNs have been used as carriers for MDR‐specific siRNAs delivery^99^ and simultaneous delivery of chemotherapeutic compounds and chemosensitizers.^106^ An in vivo study on a breast cancer xenograft showed that multifunctional MSNs can successfully deliver doxorubicin with P‐gp‐targeting siRNAs. The results showed an enhanced permeation, retention, and synergic suppression of cancer progression with substantial P‐gp downregulation.^107^ Multiple layers of doxorubicin‐ and TAT‐functionalized MSNs were self‐assembled through electrostatic interactions.^107^ Disulfide linkages were used to bind siRNAs against vascular endothelial growth factor to the outer layer. This method allowed doxorubicin to be delivered to the nucleus while siRNAs were released into the cytoplasm.^108^

#### 4.1.5 Carbon‐based nanocarriers

Nanocarriers made of carbon have been successfully employed in biological studies because of their structural stiffness, thermal expansion, profound surface‐to‐volume ratio, and capacity to modify their surfaces. Especially carbon nanotubes (CNTs) have been extensively used in cancer theranostics.^109^ CNTs have a variety of applications, including NIR fluorescent imaging tools^110^ and Raman probes due to their scattering characteristics.^111^ Single‐walled (SW) and multi‐walled (MW) CNTs are the two main categories, being altered with a variety of chemical compounds.^112^ Modified SW‐CNTs, as nanocarriers, can easily penetrate MDR cancer cells.^113^ A study on MDR pancreatic adenocarcinoma showed that paclitaxel‐plus C6 ceramide‐containing CNTs can lead to significant cellular apoptosis after imposing heat through magnetic field induction.^114^ Interestingly, MDR OVCAR8/ADR cells were successfully treated with chemotherapy using SW‐CNTs coated in hyaluronic acid with cholinic acid derivatization (CAHA).^115^ A study loaded quercetin (a P‐gp inhibitor) and N‐desmethyl tamoxifen on MW‐CNTs to distribute tamoxifen showed effective drug release into MDR MDA‐MB‐231 cells under an acidic condition.^116^ Interestingly, hollow carbon nanospheres and NIR radiation can effectively reverse MDR by producing free radicals while releasing doxorubicin.^117^ In another study, anti‐P‐gp antibody‐functionalized MWCNTs showed the ability to greatly boost phototoxicity in spheroids composed of MDR cancer cells exposed to light.^118^ For instance, siRNAs and anticancer medications have been delivered using graphene to target MDR cancer cells. Graphene oxide (GO) successfully delivered siRNAs targeting miR‐21 and adriamycin to MDR cells,^119^ and PTT in combination with chemotherapeutic drug‐functionalized GO has been utilized to treat malignancies with MDR properties.^120^, ^121^ In an in vitro study on resistant MDA‐MB‐231 cells, irinotecan and doxorubicin were added to GO, and poloxamer 188 was used to stabilize it. The produced heat could specifically activate apoptotic pathways and caused significant cytotoxicity.^122^ Another study on MDR breast cancer cells loaded two molecular beacons (MBs) and doxorubicin on a GO structure. This study demonstrated that doxorubicin was easily absorbed in a low‐pH milieu, and the two MBs could effectively silence MDR1 and erythroblastosis virus E26 oncogene homolog 1 (ETS1) mRNAs and trigger P‐gp inhibition.^123^ Before clinical administration, ad hoc structural alterations are crucial because carbon‐based nanocarriers naturally congregate, especially in physiological settings. For instance, considerable changes in graphene's chemical and physical properties can result from surface functionalization.^124^

#### 4.1.6 Silver nanoparticles

The effectiveness of silver nanoparticles (AgNPs) in managing MDR cancer depends on the NP size. However, there is a crucial window of opportunity for research that would allow focusing on some of the deadliest drug‐resistant malignancies. Gopisetty et al. investigated silver NPs size‐base cellular characteristics in MCF‐7/KCR cell.^125^ The authors found that compared to 5 nm AgNPs, 75 nm silver NPs were better at preventing P‐gp efflux activity, amplifying the apoptotic effect of doxorubicin. In contrast, the increased surface‐to‐volume ratio of 5 nm silver NPs certainly contributed to their greater efficiency as ROS generators. The endoplasmic reticulum calcium reserves were found to be affected by the 75 nm AgNPs, which led to endoplasmic reticulum stress and decreased P‐gp placement on the plasma membrane. This was thought to be the key mechanism behind P‐gp inhibition, increasing the susceptibility of breast cancer resistance to doxorubicin. AgNPs were also applied to sensitize platinum‐resistant A2780 cells to cisplatin. AgNPs and cisplatin at 8 and 62 μg/mL caused 50% of the cells to die after 48 h.^126^ Furthermore, the cumulative efflux potential of MDR cancer cells was inhibited by AgNPs when a synergistic action of AgNPs with six different anticancer drugs was examined.^127^ It has been extensively studied how to cure resistant cancers with metal NPs. In order to sensitize cancer cells, bioactive molecules, such as antibodies, can be attached to the outer sites of metal NPs like gold and silver NPs. Additionally, other chemical traits like hydrophilicity and stability also aid in the efficient delivery of drugs. Moreover, it was demonstrated that the efficacy of metal NPs depends significantly on both their size and, to a lesser extent, shape, and surface properties.^128^ More studies are required to enhance metal‐based NPs for yet more effective anticancer treatments.

#### 4.1.7 Nanodiamonds

Nanodiamonds (NDs) are up to 10 nm nanostructures consisting of tetrahedral sp^3^ hybridization. The outermost atoms of such materials must be capped in order to have standalone structures. In various hybridizations, either heteroatoms or carbon atoms can accomplish this property. NDs are amenable to functionalization techniques that can anchor various compounds. Furthermore, the presence of particular structural flaws can endow NDs with photoluminescence, facilitating PTT. In nanomedicine, these characteristics are particularly required.^129^, ^130^, ^131^ A system based on commercially available NPs was compared to NDs in terms of paclitaxel delivery into chemoresistant cells.^129^ After 48 h of culture, the effectiveness of killing carcinoma cells was improved significantly compared to nab‐paclitaxel (71% of cells were killed as compared to 40%), and it was shown that the particles quickly consolidated in lysosomes. This was a blatant indication of the NDs' improved performance in this area, most likely caused by their superior absorption and pH‐mediated drug release. It has also been reported that NDs can effectively deliver anticancer drugs by preventing the overexpressed ABC pumps in MDR cancer cells. Zhu et al. prepared NDs to administer a twofold dose of doxorubicin plus physisorbed malaridine (MAL) before layering with folate‐DOX‐PEG.^132^ The end product demonstrated a pH‐responsive cargo release, quickly distributed, and readily penetrated the cells, and through enhancing the cellular accumulation of DOX‐MAL, improved treatment effectiveness against chemoresistant MCF‐7/ADR cells. Over 90% of cells underwent apoptosis in the in vitro experiments, demonstrating the remarkable efficacy of this combinatorial strategy. However, more research is required to confirm the system's safety in vivo. Lam et al.^133^ demonstrated that NDs can also be used to reduce P‐gp efflux. A favorable therapeutic result was obtained by treating the NDs with either erlotinib or gefitinib (two anti‐EGFR inhibitors). Adding selectively targeted molecules can also improve the effectiveness of the anticancer medication. Considering mitochondrial biogenesis in cancer formation and progression, Chan et al. looked into enhancing the skin sensitivity of breast cancer cells to doxorubicin by targeting cancer cells' mitochondria.^134^ The mitochondrial localization sequences (MLSs) were employed to favor the accumulation of drugs in mitochondria over proteolytic enzymes to further improve performance. As a result, a superior system was attained. This approach can improve the efficacy of novel immunotherapeutic agents (such as anti‐programmed death protein‐1) and radiotherapy by targeting the cancer cells' mitochondria.^135^, ^136^, ^137^

#### 4.1.8 Graphene and graphene oxide

A single honeycomb lattice makes up the 2D substance known as graphene. This substance is electrically conductive with a high surface area (~two times greater than CNTs). From ultraviolet through NIR, fluorescence is seen. PTT or thermoacoustic therapy is possible when light or acoustic waves are absorbed and release heat. However, it has a large number of delocalized electrons, which limits the usefulness of the material and causes an extemporaneous aggregation of complex graphene planes. Therefore, GO is more commonly used in its oxidized state. GO has been utilized to target a variety of malignancies, including chemoresistance and metastatic cancers. In a study, Pei et al. transported a combination of two chemotherapeutic medications (paclitaxel and doxorubicin) into the cancer cells using GO nanocarriers. This procedure led to an efficient eradication of tumors, both in vitro and in vivo, without producing significant side effects.^138^ Furthermore, Tiwari et al.^139^ and Tran et al.^122^ used a similar strategy of mixing more than one medicine with promising outcomes. In an investigation, Thapa et al.^140^ applied a PEGylated lipid bilayer‐wrapped nano‐GO containing doxorubicin coupled with rapamycin, an inhibitor of the cell survival pathways PI3K/Akt/mTOR, to reduce the resistance of cancer cells. The chemical and PTT effectively killed around 80% of chemoresistant cancer cells. In different studies, adding drug‐targeting molecules against lactobionic acid,^141^ hypericin,^142^ prostate stem cell antigen,^143^ or hyaluronic acid^144^ led to more targeted and efficient cancer eradication. Interestingly, Guo et al. used magnetic particles to obtain high resolution and contrast imaging of malignancies.^143^ These therapies make the cancer cells more receptive to additional therapies, allowing for the efficient delivery of higher therapeutic agent doses while reducing the possibility of unfavorable side effects.^145^

### 4.2 Organic nanocarriers

**FIGURE 3:**
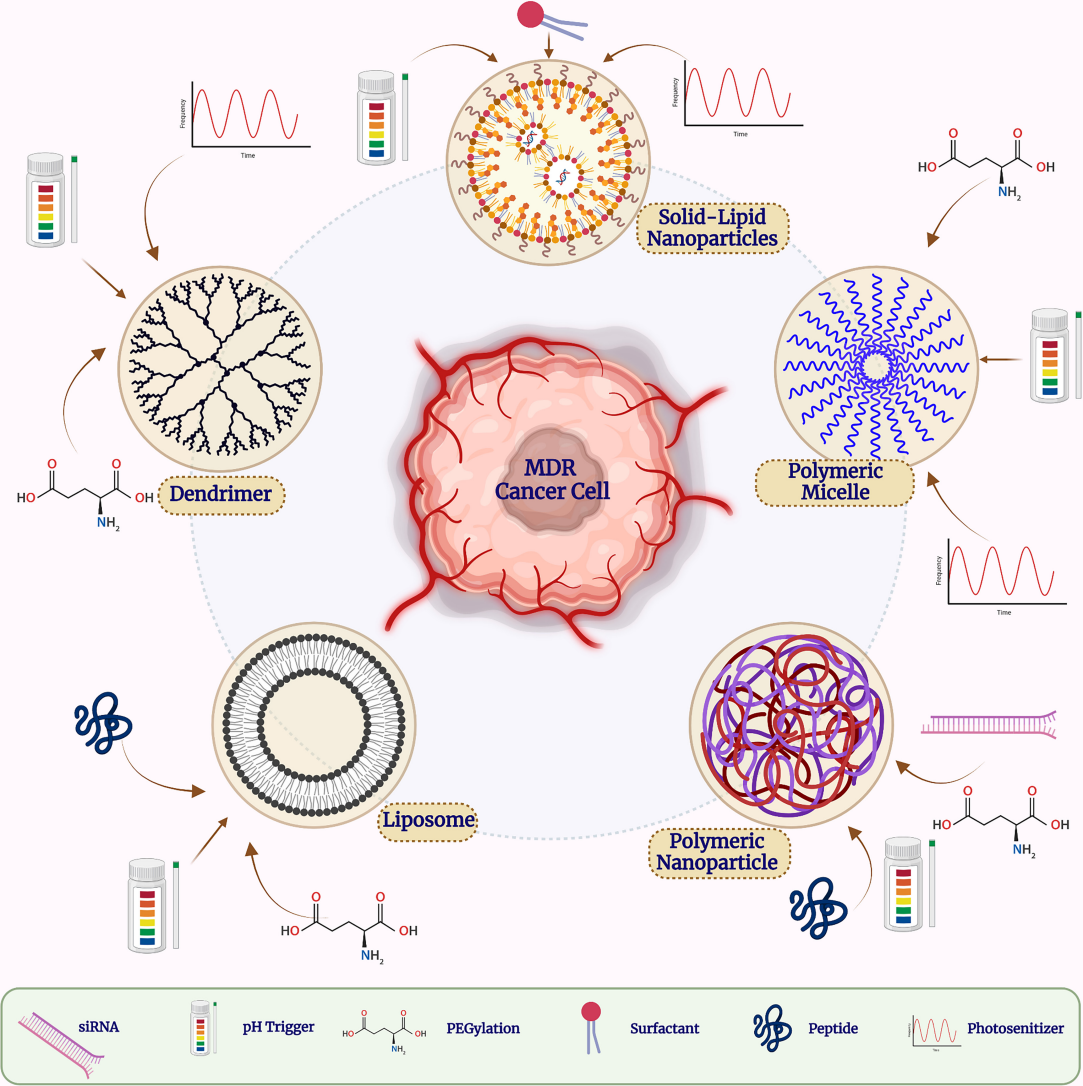
An illustration of the primary organic nanoparticles under investigation for preventing MDR in cancer. Various polymeric and lipid‐based NPs are shown from the left to the right side. There have been reports of potential functionalization, including targeted ligands and external stimuli used to initiate drug release. MDR, multidrug resistance; SLN, solid lipid nanoparticles; NLCs, nanostructures lipid carriers.

Organic NPs have some benefits, including biocompatibility and biodegradability, compared to inorganic NPs. They are highly adaptable as they may be made of amphiphilic molecules. Like inorganic NPs, they can significantly change for a specified distribution. This section demonstrates how these nanomaterials can successfully inhibit the MDR properties of cancer cells **(**Figure 3 **)**.

#### 4.2.1 Lipid‐based nanoparticles

Liposomes are made up of an aqueous core and phospholipid bilayer. They are exceedingly adaptable and may enclose both hydrophilic and hydrophobic compounds.^146^ Liposomes are minimally cytotoxic and biocompatible since they typically comprise natural phospholipids.^147^ Drug stability is improved by the capacity to encapsulate them in liposomes, preventing circulating drug breakdown,^148^, ^149^ and PEGylation to increase their stability and circulation times.^150^ Liposomes can target tumor cells specifically and uniformly due to surface functionalization. Pegylated liposomes co‐loaded with paclitaxel and resveratrol effectively inhibited tumor development in mice by inducing MDR‐cytotoxicity in mammary cancer cells as well as bioavailability and retention.^151^ Interestingly, some phospholipids have been shown to directly prevent the activity of P‐gp.^152^ Compared to the individual medications, liposomes comprising doxorubicin and carfilzomib displayed better MDR inhibition. This combination has resulted in a significant reduction in tumor volume during both in vitro and in vivo studies on myeloma cells.^153^ Recently, liposomal Vyxeos™ was developed to deliver daunorubicin and cytarabine for acute myeloid leukemia.^154^ Notably, vesicular systems, such as liposomes, niosomes, transfersomes, ethosomes, and modified vesicular systems (surface functionalized liposomes), can respond to external stimuli for site‐specific drug delivery of chemotherapeutics.^155^ Assanhou et al. demonstrated that a dual‐functionalized cationic liposome containing 1,5‐dioctadecyl‐N‐histidyl‐L‐glutamate, a synthetic cationic lipid, can facilitate the intracellular accumulation and mitochondria targeting of paclitaxel. These NPs can effectively stop tumor growth.^156^ Wax, fatty acids, and glycerides are examples of solid lipids at the body temperature and form solid lipid nanoparticles (SLNs), which can also include a stabilizing surfactant as created.^157^ Although their crystallinity can lead to poor drug loading, they have superior drug stability and extended‐release than liposomes. Encapsulated paclitaxel plus verapamil can prevent drug efflux and chemoresistance in breast cancer cells.^158^ Lipid NPs loaded with curcumin and doxorubicin enhances the cytotoxicity rate. Experiments on hepatocellular cell lines and mouse models demonstrated that this combination achieves MDR evasion at lower dosages.^159^ Despite this efficacy in several cancer lines, K‐562 (a leukemic cell line) showed no cytotoxicity to synthetic lipid edelfosine. Nanoencapsulation caused cancer cells to die through autophagy pathways, and following endocytosis, there was a noticeable increase in the number of autophagic vesicles, potentially combating MDR in this cell line.^160^ It has been demonstrated that SLNs can be pH‐responsive and release doxorubicin when loaded with the drug in the MDR cells' acidic microenvironment.^161^ Despite these advances, there are still significant structural obstacles related to the crystallinity of SLNs. As a result, recently developed nanostructured lipid carriers made of liquid lipids have been developed.^162^ Higher payloads can be packed into the partly crystalline solid matrices, increasing medication availability. These NPs have been considered potential drug‐delivery devices in treating MDR malignancies. For example, nanostructured lipid carriers (NLCs) were created to deliver indocyanine green and paclitaxel for combination chemotherapy in accordance with photodynamic treatment (PDT). While the laser‐based irradiation enhanced drug release, ROS formation, and increased cytotoxicity, the nanoformulation enhanced the drug stability. Additionally, NPs successfully targeted the tumor cells in selected mice, offering great promise for cancer treatment.^163^ Doxil (liposomal product of doxorubicin) was promised to be safe and compatible with the expected therapeutic outcome. However, inadequate nuclear delivery of doxorubicin following cellular uptake (<0.4%) has hindered its generalized application.^164^ Zhu et al. found that doping a small amount of PpIX molecules to the lipid bilayer of chemodrug‐bearing liposomes can enhance the nuclear delivery of doxorubicin.^164^

#### 4.2.2 Polymeric nanoparticles

Polymeric NPs have been considered versatile nanomaterials for transporting medicines and macromolecules and possible methods for reversing MDR in cancer.^151^ As an illustration, an in vivo study on the MDR colon cancer murine model indicated that paclitaxel‐based NPs exhibited higher toxicity and fewer toxicities than paclitaxel.^165^ Paclitaxel and fluorouracil nanostructures were more cytotoxic than either treatment alone and effectively reversed MDR.^166^ Polymeric NPs can respond to external stimuli and are highly adaptable. For instance, acetylated cyclodextrin‐based pH‐responsive nanocarriers have demonstrated enhanced efficiency of numerous anticancer drugs in MDR cancers.^167^ Remarkably, peptide‐linked chains responsive to TME matrix metalloproteinases (MMPs) have been used to deliver anticancer medicines.^168^ Recent research has led to the developing a selective copolymer matrix (PEG2k‐pp‐PE) that is highly sensitive to MMP2 and capable of inhibiting P‐gp, reversing MDR.^169^ An in vivo study demonstrated that vincristine‐ and quercetin‐loaded lipid polymeric hybrid NPs have a prolonged release profile and cytotoxicity in an MDR non‐Hodgkin lymphoma model.^170^ These NPs have been successfully engineered to better interfere with MDR processes. An experimental study showed that MDR in breast cancer cells was effectively overcome by a self‐assembled class of NPs delivering shRNA and doxorubicin directed at the *Survivin* gene (an apoptosis inhibitor).^171^ To target cancer cells, numerous approaches have utilized PLGA (poly lactide‐co‐glycolide), a class of biodegradable polymers with surface modifications.^172^, ^173^ By targeting in vitro and xenografted CD44^+^ ovarian cancer cells, PLGA nanoparticulate systems comprising FAK (focal adhesion kinase)‐targeting siRNA and paclitaxel could successfully reduce MDR.^174^ To deliver vincristine sulfate and overcome MDR in breast cancer cells, polymer‐based NPs that are made up of cell‐penetrating peptide R7 conjugated with PLGA‐PEG (an acid‐sensitive PLGA‐PEG‐folate), was developed.^175^ An experimental study loaded docetaxel and poloxamer 235 on PLGA–TPGS NPs. Well‐known co‐polymers, called poloxamers, can block P‐gp efflux pumps and reverse MDR. In this in vivo study on MDR human breast carcinoma, the designed NPs were effectively taken up by cells, demonstrating their significant potential for clinical use.^176^ When PLGA NPs were loaded with SN‐38 and treated with d‐tocopheryl PEG 2000 succinate (TPGS2k), MDR A549 cells experienced cytotoxicity. Interestingly, the designed drugs could interact with the structure and operation of mitochondria after release, decreasing the production of ATP and ultimately hindering P‐gp efflux pumps.^117^ Although podophyllotoxin is renowned for P‐gp inhibition, using it in a clinical setting is challenging due to its poor solubility and high toxicity. Therefore, it is selectively gathered within tumors and enhances effectiveness in MDR cancers in vivo after conjugation with CMC‐Ac (acetylated carboxymethyl cellulose) and PEG.^177^

#### 4.2.3 Polymeric micelles

These amphiphilic copolymer‐based nanomaterials pose significant hydrophilic covering and a hydrophobic inside core where pharmaceuticals can be readily enclosed.^178^ Hydrophobic pharmaceuticals are frequently enclosed in amphiphilic block copolymers made by Pluronic®.^179^ Notably, Pluronic® has been demonstrated to be ingested by endocytosis to localize inside the mitochondria and to interfere mitochondrial electron transport chain, reducing ATP content of MDR cancer cells.^180^ Literature shows that Pluronic® P85 can decrease cellular ATP content, particularly in MDR cancer cells, accompanied by reduced P‐gp levels.^181^, ^182^ Micelles containing siRNAs and paclitaxel targeting PLK1 (polo‐like kinase 1) can successfully deplete ATP content and reduce the cancer cells' chemoresistance.^183^ This effect is possibly through inactivating the ATP‐dependent P‐gp efflux pumps.^45^ When given to MDR cancer cells, micelles containing hydrophilic natured PEG and hydrophobic polyphosphoester blocks could effectively release doxorubicin. Micelles disassemble when the disulfide bond breaks, resulting in sustained resistance. Similar outcomes were attained in mouse xenografts, supporting its potential therapeutic applicability.^184^ A polymer‐based micelle has been indicated to grow larger in acidic environments and shrink in the presence of high intracellular glutathione. Doxorubicin was successfully delivered to the MDR breast cancer cells' nucleus by this system.^185^ Yu et al.^186^ created an intriguing theranostic system consisting of NIR and pH‐responsive polymer‐based micelles containing a prodrug of doxorubicin disintegrated in an acidic environment. The investigators found that the permeation and release of drugs into MDR breast tumor cells (in both in vitro and in vivo settings) were improved using NIR irradiation (hyperthermia). The same team created micelles, including a doxorubicin polymeric prodrug, a pH‐sensitive copolymer, and a photosensitizer. In an acidic environment, the nanomaterial activated fluorescence signals, and following NIR radiation, ROSs were produced, and doxorubicin was released, resulting in PDT. After that, NIR light was transformed into heat to contribute to MDR tumor photothermal therapy, photoacoustic imaging, and drug penetration.^187^ Both folate receptor‐mediated internalization of micelles and external alteration of pH‐responsive micelles have enabled active cancer cell targeting. It has been established that these approaches can impede P‐gp activity in human breast carcinoma cells, enhancing medication absorption and retention and eventually combating MDR. This process happens when the drug candidate is released from endosomes and affected by low pH.^188^, ^189^, ^190^

#### 4.2.4 Dendrimers

Polymers with a dendritic backbone are organized to design a spherical shape to build up dendrimers. They offer higher stability and less cytotoxicity by delivering medications that are adhered to their surface or enclosed in the created cavity.^191^ A study combined doxorubicin with a dendrimer bearing phthalocyanine and intermingled with a micelle made of poly (ethylene glycol)‐b‐poly(L‐lysine), a class of block copolymer (PEG‐PLL). This structure was then endocytosed and collected in vesicular compartments after radiation exposure. The study showed that doxorubicin was localized inside nuclei in MDR MCF‐7 cells and also in a xenograft model.^192^

### 4.3 Hybrid nanoparticles

While each NP category has its own benefits and limitations, combining organic and inorganic NPs can result in a hybrid drug delivery system that boasts superior biological properties, resulting in improved treatment effectiveness and reduced drug resistance.^193^ Hybrid nanoparticles that combine lipids and polymers, featuring a lipid shell and a polymeric inner core, show great potential in the treatment of metastatic prostate cancer,^194^ breast cancer,^195^, ^196^ and pancreatic cancer.^197^, ^198^ Hybrid nanoparticles have the ability to contain both hydrophilic and hydrophobic medications, resulting in improved therapeutic outcomes achieved by combining the favorable biocompatibility of lipid compounds with the structural stability of polymer NPs.^199^, ^200^ The reticuloendothelial system does not quickly remove this system, which allows cancer cells to ingest it effectively.^201^, ^202^ A common method for producing NPs is to combine organic and inorganic materials. It has been shown that liposome–silica hybrid (LSH) NPs are capable of delivering drugs to prostate and breast carcinoma cells. These NPs consist of a silica core surrounded by a compatible lipid bilayer.^203^ In a pancreatic cancer murine model, gemcitabine and paclitaxel have been reported to be delivered to cancer cells synergistically using the LSH NP.^204^ By combining giant liposomes and porous silicon NPs onto a microfluidic chip, Kong et al. created a cutting‐edge nano‐in‐micro platform. Co‐delivering drugs and synthetic DNA nanostructures through this platform can significantly boost the cell death rate of doxorubicin‐resistant breast cancer cells.^205^ In a different research, a combination of CNTs and chitosan NPs was used to transport methotrexate to lung cancer cells. Results indicated that this delivery method may enhance the effectiveness of the anticancer treatment while reducing the harmful effects on healthy cells.^206^ Additionally, PLGA hybrid NPs and half‐shells inside metal multilayers comprising gold and manganese offer the potential to combine targeted drug release with hyperthermia that might augment the devastation of cancer cells.^207^ Kong et al. developed a cutting‐edge nano‐in‐micro platform by fusing enormous liposomes with porous silicon NPs on a microfluidic device.^205^ It has been demonstrated that using this technology to deliver medicines and artificial DNA nanostructures simultaneously greatly accelerates the killing of doxorubicin‐resistant breast cancer cells. According to Parodi et al.'s study,^208^ one way to prevent phagocytes from removing nanocarriers is to cover nanoporous silicon‐based particles with a leukocyte‐purified cell membrane. This hybrid NP prolongs the drug's circulation to increase intratumoral accumulation. Similarly, several other studies used mesoporous silica NPs (MSNPs) covered in cancer cell membranes to treat cancer, refining stability and targeting the power of nanocarriers.^209^ Additionally, developing NPs with dual‐membrane coating can improve their functionality even more. For instance, membrane‐coated NPs using erythrocyte‐platelet hybrid and erythrocyte carcinoma hybrid coatings have improved stability and extended circulation life.^210^, ^211^, ^212^

In recent decades, the EPR targeting approach has been a key method for targeting nano‐chemotherapeutics. However, there have been some challenges when using nano‐chemotherapeutics with low molecular weight. In these cases, nano‐chemotherapeutics tend to reenter the bloodstream through diffusion. The decreased duration of tumor residence resulting from this phenomenon underscores the importance of enhancing the nano‐targeting of chemotherapeutic agents. This can be achieved by considering the evolving pathophysiological properties of tumoral tissues, as Din et al. noted.^213^ Conventional chemotherapy's limitations have prompted the development of nano‐chemotherapeutics that target TME specifically. This approach has shown promise in combating drug resistance.^213^ Zhang et al. have recently shown that using TME‐responsive nanomedicine is a safe and effective way to diagnose and treat tumors. The in situ biosynthesis of inorganic nanomaterials within tumors in response to specific TME targets can offer several benefits, including evading scavenging by the innate immune system and enhancing intratumoral accumulation, collectively improving the cytotoxicity.^214^ Song et al. suggested utilizing nanomaterials to modify the TME in order to improve the effectiveness of sonodynamic therapy (SDT). This is a noninvasive therapeutic approach using sonosensitizers to generate ROS, inducing tumor cell death and boosting the immune response. This modality is considered more favorable than PDT due to its ability to penetrate deeper into tissues.^215^ Zhang et al. demonstrated the use of nanomedicine in regulating cancer and inflammation. They also discussed the progress and rationale behind nanobiotechnology and the corresponding therapeutic strategies in modifying pyroptosis to treat cancer and other inflammation‐related diseases. Pyroptosis exhibits a strong correlation with both neoplastic growths and inflammatory pathologies.^216^

For efficient administration of nano‐chemotherapeutics to the TME, one must consider its intrinsic factors, including hypoxia, acidosis, elevated interstitial fluid pressure, enzymatic activity, oxidative stress, hyperthermia, inflammation, and diverse levels of amino acids, proteins, and DNA fragments.^217^ In preclinical studies, several strategies have been implemented, such as surface functionalization (PEGylation), stimuli‐responsive NPs, hybrid NPs, and dual functional carriers, and executed efficacious outcomes to treat cancer by targeting TME. These strategies are PEGylation,^218^ small‐size NPs, reversing the surface charge, generation of CO_2_ using hyperthermia,^219^ pH‐responsive NPs,^220^ temperature‐responsive NPs,^221^ or NPs responsive to different physical triggers, including magnetic field,^222^ light,^223^ ultrasound.^224^ There are several classifications for nano‐chemotherapeutics that target the TME. These classifications are based on different materials such as polymers, carbon, lipids, surfactants, silica, metals, or metal oxides. Scientists have created nano‐sized chemotherapy treatments that specifically target the acidic environment found in tumors. They achieve this by using acid‐sensitive polymers like polyethyleneimine‐Schiff base, poly(styrene‐co‐maleic anhydride), poly(beta‐amino ester), and poly(2‐(diisopropylamino)‐ethylmethacrylate). Table 1 outlines the indexed studies applying NPs to overcome drug resistance in different cancers.^89^, ^225^, ^226^, ^227^, ^228^, ^229^, ^230^, ^231^, ^232^

**TABLE 1:** A summary of the most important research on nanomedicines that aim to combat cancer's multidrug resistance. (Adapted from Martinelli et al. study).^225^

Nanoparticle	Anticancer drug	Ligand moiety	Cancer type	Outcome	Ref.
Polymeric nanoparticles	Vincristine sulfate	Folate & R7 Peptide	MDR breast cancer	Antitumor efficacy *(*in vivo*)* Increased cytotoxicity (in vitro*)*	[157]
	Paclitaxel	siRNA‐ Focal Adhesion Kinase	MDR ovarian cancer	Reduced tumor development (in vivo*)*, Anticancer activity & apoptosis (in vitro*)*	[156]
	Doxorubicin	shRNA Survivin	MDR breast cancer	Cytotoxic effects Suppression of P‐glycoprotein and glutathione S‐transferase *(*in vitro*)* Decreased survivor protein (Survivin) Activity	[232]
Polymeric micelles	Doxorubicin	Folate	MDR ovarian cancer	Reduced tumor proliferation *(*in vivo*)* Improvements in cytotoxic effects (in vitro*)*	[171]
	Doxorubicin	Folate	MDR breast cancer	Reduced tumor proliferation *(*in vivo*)* Improvements in cytotoxic effects (in vitro*)*	[169]
	Paclitaxel	siRNA‐Polo‐like kinase‐1	MDR colorectal cancer	The combination of ATP depletion & PLK1 suppression reduced tumor growth (in vitro *and* in vivo*)*	[229] [165]
Liposome	Paclitaxel	Mitochondrial Targeting Peptide	MDR lung cancer	Reduced tumor proliferation (in vivo*)* accelerated apoptosis (in vitro*)*	[231]
Graphene oxide	Doxorubicin	Erythroblastosis Virus E26 Oncogene Homolog‐1 & Multidrug Resistance	MDR breast cancer	Downregulation of Erythroblastosis Virus E26 Oncogene Homolog‐1 & multidrug resistance	[124]
	Adriamycin	siRNA miR‐21	MDR breast cancer	Improvements in cytotoxic effects (in vitro*)*	[230]
Multi‐walled carbon nanotube	‐	Anti‐P‐glycoprotein antibody	MDR ovarian cancer	Profound phototoxicity in cancer cell entities	[229]
Gold nanoparticles	Doxorubicin	Anti‐DR‐4 antibody	MDR colorectal cancer	NIR radiation slows rate of tumor development	[89]
	Thymoquinone	siRNA Akt	MDR breast cancer	Down‐regulation of Akt (in vitro) correlates with increased cytotoxicity (in vivo*)*	[226]
Mesoporous silica nanoparticles	Doxorubicin	siRNA‐P‐Glycoprotein	MDR breast cancer	The decreasing the expression of p‐glycoprotein (in vivo*)*	[228]
CdSe/ZnSe quantum dot	Doxorubicin	siRNA‐P‐Glycoprotein	MDR cervical cancer	The decreasing the expression of p‐glycoprotein (in vivo*)*	[227]
Iron oxide nanoparticles	Doxorubicin	siRNA‐P‐Glycoprotein	MDR breast cancer	The decreasing the expression of p‐glycoprotein *(*in vivo and in vitro*)*	[225]

Abbreviations: ETS1, erythroblastosis virus E26 oncogene homolog 1; FAK, Focal adhesion kinase; GO, graphene oxide; GST, glutathione S‐transferase; MDR, multidrug resistance; MWCNT; Multi‐walled carbon nanotubes; NIR, near‐infrared; P‐gp, P‐glycoprotein; PLK1, polo‐like kinase 1; QD, quantum dot; siRNA, small interfering RNA.

## 5 HOW NANOPARTICLES OVERCOME DRUG RESISTANCE?

Despite advances in cancer‐targeting agents, drug resistance is still a significant concern leading to cancer recurrence. The overexpression of ABC transporters (such as the efflux transporter),^233^ malfunctioning apoptotic pathways, high interstitial fluid pressure, acidosis, and hypoxia are just a few examples of the physiological and cellular factors that lead to tumor drug resistance. It has been demonstrated that nanotechnology used in drug delivery for cancer treatment can significantly help overcome drug resistance. In order to combat MDR, considerable efforts have been dedicated to developing therapeutic approaches that work together in synergy. These involve using MDR blockers or combining chemotherapy with other treatments like gene therapy, phototherapy, and gas therapy. The goal is to sensitize drug‐resistant cells to chemotherapy. Particularly, nano‐based drug delivery systems have demonstrated the ability to combine several drug candidates into a single system. The review discussed the recent advancement of nano‐based strategies with the dual goals of enhancing chemotherapy effectiveness and overcoming the MDR of cancer. The detailed description of the benefits and underlying mechanisms of action of the different combinatorial techniques was provided, and it was then discussed how these strategies can assist in combating MDR. In clinical settings, the emergence of MDR in cancer patients due to repeated chemotherapy has become a major reason for tumor recurrence. At the genetic and molecular levels, the core mechanism to address how cancerous cells evolve to become drug‐resistant is exceedingly complex and difficult. However, via the use of anticancer medications, biologists have identified some key mechanisms that cancer cells use to survive.^234^

### 5.1 Targeting drug efflux transporters

Efflux transporters are members of the ABC transporter family, mediating drug resistance. The drug is pumped out of the cell by the overexpressed efflux transporters, which lowers intracellular drug concentration and results in treatment failure.^235^ In many tumors, including breast and ovarian cancers, over‐expression of P‐gp is linked to poor treatment outcomes.^236^ Numerous investigations have established that some chemotherapeutic drug‐loaded NPs can avoid the exposure of antitumor drugs to efflux transporters by enabling the release of candidate drugs at the perinuclear site, away from the cellular membrane and efflux pumps. NPs can enter cells primarily through endocytosis rather than diffusion.^237^ The drug release control can be changed by NP‐based drug delivery technology. For instance, some studies used oxidative stress and acidosis as triggers for the release of drugs from NPs.^238^ Additionally, polymeric NPs can function as MDR modulators.^239^ For example, micelles developed with N‐(2‐hydroxypropyl) methacrylamide (i.e., HPMA) as well as poly (propylene oxide) co‐block (PPO) are capable of inhibiting P‐gp.^240^ Another method for treating MDR cancers is combination therapy. A combination therapy using NP has been effective in addressing the issue of pharmacokinetic differences among drugs, combating drug resistance, and enhancing the effectiveness of cancer treatment by combining multiple therapeutic ingredients in a single drug carrier.^241^, ^242^, ^243^, ^244^ Another approach to combat drug resistance caused by efflux transporters would be prohibiting their generation and function. This strategy can be carried out by either developing NPs containing both chemotherapeutics and efflux pump inhibitors or reducing the quantity of ATP provided to the efflux pump.^245^ Because cyclooxygenase‐2 (COX‐2) can contribute to P‐gp‐related MDR, a highly selective COX‐2 inhibitor may decrease the P‐gp expression.^241^ According to a recent study, the MDR of breast carcinoma cells was overcome by the co‐delivery of typical COX‐2 inhibitors and doxorubicin using NPs.^245^ In addition, numerous studies have demonstrated that P‐gp‐targeted siRNA and chemotherapeutics delivered by NPs can improve treatment response by repressing the expression of ABC transporters. A recent study showed that the combination of doxorubicin and miRNA‐495 with a silica nanoparticle coated with the carcinoma cell membrane can effectively overcome lung cancer MDR. This study showed that miR‐495 dramatically decreased P‐gp expression in drug‐resistant tumor cells.^246^

### 5.2 Targeting the apoptotic pathway

Drug resistance in cancer can result from defective apoptotic machinery, which enhances cancer cells' survival.^247^ Deregulation of Bcl‐2 and associated nuclear factor comprising kappa B (NF‐kB) can defect the apoptotic pathway. Bcl‐2 is a renowned antiapoptotic mediator that contributes to drug resistance. This interaction implies Bcl‐2 can be an appropriate target to overcome chemotherapy resistance. To this end, several studies applied the co‐delivery of Bcl‐2‐targeting cyclic siRNA and chemotherapeutic agents via NPs.^248^, ^249^, ^250^, ^251^ Besides, NF‐kB inhibitors like curcumin and pyrrolidine dithiocarbamate (PDTC) were explored in NP‐based combination treatment. The proapoptotic mediators can be triggered to inhibit antiapoptotic moieties and restart the apoptotic signaling pathways. For instance, conjoining ceramide with paclitaxel advanced the therapeutic efficacy of numerous tumor models resistant to conventional chemotherapy.^252^, ^253^ A recent study discovered that ceramide might control alternative pre‐mRNA splicing to restore the expression of the wild‐type p53 (a tumor suppressor). NPs provide a more efficient platform for delivering ceramide into cancer cells carrying p53 missense mutation.^254^ Restoring the role of p53 or other tumor suppressors is a promising strategy for contending cancer medication resistance since p53 offers a substantial role in apoptosis. As a result, more studies have been done on p53 gene remedies using an NP‐based delivery system. It has been demonstrated that cationic‐type solid lipid NPs and PLGA have been shown to transfect the p53 gene in lung^255^ and breast cancer cells,^256^ respectively. These findings establish a potent apoptosis induction and tumor growth inhibition.

### 5.3 Targeting hypoxia

Another TME feature that contributes to MDR is hypoxia.^257^ Cancer cells often exist in hypoxic conditions due to abnormal blood vessels and higher oxygen requirements for rapid proliferation. Tumor hypoxia can lead to chemotherapy resistance in several ways. For example, slowly proliferating cells in hypoxic areas can avoid being destroyed by cytotoxic chemotherapy drugs like alkylating agents. Hypoxia also creates an oxygen gradient inside the tumor, which boosts tumor heterogeneity and encourages more aggressive traits. Furthermore, hypoxia can enhance the expression of drug efflux glycoproteins.^257^ In this condition, hypoxia‐inducible factor‐1α (HIF‐1α) is overexpressed and conducts the expression of genes responsible for MDR.^258^ As a result, another strategy to overcome drug resistance is to target HIF‐1α.^259^ The use of NPs in the management of hypoxia has also been comprehensively studied. One method to prevent a hypoxic environment is to silence the *HIF‐1α* gene. Several studies have shown that nanosystems containing HIF‐1α siRNA can effectively help to overcome chemotherapy resistance.^260^, ^261^ Drug resistance brought on by hypoxia has also been therapeutically reduced by HIF‐1α inhibitors.^262^ HIF‐1 signaling can be inhibited indirectly or indirectly; for example, PI3K/Akt/mTOR signaling pathway can control the expression of HIF‐1a, and by inhibiting this pathway, HIF‐1 α expression is downregulated, increasing MDR cells' sensitivity to cancer treatments.^263^ In this process, sets of NPs can provide superior platforms to achieve combination therapy, such as PEGylated and non‐PEGylated liposomes.^264^ Moreover, HIF‐1 transcriptional potential depends on heat shock protein 90 (HSP90). It has been demonstrated that HSP90 inhibition can suppress HIF‐1 α expression.^265^ Loading an HSP90 inhibitor into 17AAG‐loaded NPs can considerably improve the therapy of bladder cancer.^266^

### 5.4 Bacteria‐induced drug resistance in cancer

Bacteria can develop acquired drug resistance through genetic, mechanical, or either mechanisms. For instance, through protein modification of receptors or other biologic targets, improving drug efflux pumps, or impeding drug influx. Throughout medical history, there have been many instances where antibiotics have played a similar role. The WHO (World Health Organization) has identified drug resistance as one of the top three challenges facing public health in the twenty‐first century. The issue of bacterial antibiotic tolerance is an ongoing and growing matter in the field of clinical science. Bacteria have a shorter lifespan than eukaryotic organisms, making them more sensitive to genetic variation and evolution.^267^, ^268^ Zhang et al.^269^ developed self‐traceable nanoreservoirs with simultaneous loading of gemcitabine and ciprofloxacin fabricated with hyaluronic acid for active tumor targeting. The nanoreservoirs are able to target tumor cells directly, eliminate intratumoral bacteria, and hinder the growth of tumors, all without causing any noticeable harm to healthy tissues. In a study, the treatment of the bacterial‐infected tumor with nanoreservoirs resulted in a notable increase in mature dendritic cells. This was due to the effect of the dead bacteria within the tumor that were killed by ciprofloxacin. Additionally, the presence of gemcitabine in the nanoreservoirs caused a decrease in the level of immunosuppressive myeloid‐derived suppressor cells (MDSCs).^269^

### 5.5 Strategies for overcoming cancer drug resistance via nanosystems

Nanomedicine possesses superior properties compared to small‐molecule chemotherapeutic agents, including prolonged circulation time, more intratumoral storage, fewer adverse effects, and, contribution to reverse drug resistance. As chemotherapy with only one chemodrug often does not yield satisfactory therapeutic outcomes, numerous synergistic nano strategies have been developed for delivering multiple chemodrugs or a combination of agents with diverse approaches. These cutting‐edge techniques can work together to increase the cellular demise of multidrug‐resistant tumor cells by blocking drug efflux carriers, controlling the ROS level, interrupting energy consumption, and inducing cancer cell death by targeting various pathological signaling pathways.

#### 5.5.1 Gene delivery and chemotherapy via nanosystems

It is common practice to deliver therapeutic nucleic acids inside tumor cells in order to stop the MDR signaling pathways. RNA interference (RNAi), a biological procedure capable of suppressing gene expression, is frequently used in this approach.^270^ The transfer of siRNAs (small interfering RNAs), shRNAs (small hairpin RNAs), and miRNAs (microRNAs) inside tumor cells, are different types of gene therapies based on RNA interference (RNAi).^271^, ^272^ The use of RNAi technology has been limited due to reduced cellular absorption and inadequate biological stability of RNAs. To address these issues, many nanocarriers have been designed to enhance RNA stability in circulation, facilitate their accumulation, and increase cancer cell absorption.^273^ Moreover, scientists are involved in creating diverse responsive nanocarriers that can precisely release their contents when specific pathological biomarkers are present. These smart nanocarriers are beneficial in transporting genetic materials with improved tumor selectivity and enhanced biosafety.^274^

#### 5.5.2 Delivery of pooled siRNAs

Drug‐resistant tumors have garnered a lot of interest in the co‐delivery of anticancer medications with siRNA. Co‐delivery methods have the potential to enhance the effectiveness of chemotherapy drugs by simultaneously inhibiting both efflux pump other mechanisms of MDR in cancer cells. Besides, simultaneous administration of anticancer drugs and siRNAs can silence the genes responsible for drug resistance, drug efflux pumps, and antiapoptosis pathways. This can help increase the effectiveness of the anticancer drugs.^277^ An example of this approach is the successful combination of a cisplatin prodrug and multiple siRNAs within NMOFs (nanoscale metal–organic frameworks) to achieve superior results in chemoresistant ovarian cancer cells.^275^ The siRNAs utilized for gene‐silencing were against *P‐gp*, *Bcl‐2*, and *Survivin* genes. In this study, the applied NMOFs could effectively shield the siRNAs from degradation by nucleases. Additionally, they improved the uptake of siRNAs into cells and facilitated their endosomal escape, ultimately reducing MDR gene expression. Overall, the NMOFs loaded with both siRNA and cisplatin demonstrated remarkable efficacy in gene silencing and cytotoxicity in drug‐resistant cancer cells. In 2014, Fatemian and colleagues utilized a versatile mesoporous silica nanoparticle (MSNP) transporter to overcome doxorubicin resistance in an MDR breast cancer xenograft. They achieved this by co‐administering both doxorubicin and siRNA targeting the P‐gp drug exporter.^276^ In 2017, Roberts et al. applied a dual NP‐delivery system to overcome chemoresistance in ovarian cancer cells. In this study, the investigators applied polyamidoamine (PAMAM) dendrimers and MSNs for intracellular delivery of TWIST siRNA.^278^ In 2021, Yuan et al. designed specific nanocarriers able to effectively shield doxorubicin and GCN5‐targeting siRNA, preventing premature leakage. This hyaluronic acid‐coated nanocarrier was designed to release doxorubicin and siRNA intracellularly in response to pH changes and oxidative processes.^279^

#### 5.5.3 Synergistic effects of gas therapy and chemotherapy

Gas therapy has gained significant attention as a potential treatment for cancer in recent years.^280^ Ding et al. (2019)^281^ designed a core‐shell structured nanocomposites‐based NIR light‐responsive nitric oxide (NO) delivery nano‐based platform for targeting MDR tumors. This combined treatment of gas, chemotherapy, and photothermal therapy not only shows a strong and complementary effect in fighting multidrug‐resistant cancer but also offers an effective method for creating a versatile nano‐drug delivery system with various therapeutic approaches. Tian et al. (2017)^282^ initially formed a polymeric core by encapsulating doxorubicin (DOX) and hemoglobin (Hb) in PLGA and then coated it with a cancer cell membrane and DSPE‐PEG (1,2‐distearoyl‐sn‐glycero‐3‐phosphoethanolamine‐N‐[amino(polyethylene glycol)]) for homologous targeting. The resultant DHCNPs (DOX/Hb‐based PLGA‐cancer cell membrane NPs) could effectively deliver O_2_ to cancer cells and downregulate P‐gp and HIF‐1. This effect further increased the doxorubicin accumulation inside cancer cells. Emerging studies have applied gas‐generating nanoplatforms for targeted delivery and controlled release of drugs.^283^ By lowering P‐gp expression, the molecular messenger nitric oxide (NO) can counteract the MDR effect of cancer cells, which helps foster a favorable milieu for the treatment of doxorubicin‐resistant cancer cells. One way to overcome challenges presented by multidrug‐resistant (MDR) tumors and achieve successful treatment outcomes for chemoresistant tumors is to create advanced nanosystems that can release NO and chemodrug in a controlled manner.^284^ Liu et al. (2021)^285^ created polymersomes that contain NO and have a high level of NO donors attached to the polymer chains, which helps maintain stability. IR780 iodide (a photosensitizer) and DOX‐HCl (a chemodrug) can both be concurrently injected into these polymersomes' lumen and membrane layers. The release of NO can be induced by reduction conditions and further enhanced by remote NIR light by increasing local temperature. Instantaneous high‐concentration NO release effectively reduces P‐gp expression and increases chemotherapeutic sensitivity, overcoming MDR. In order to successfully treat cancer, Wu and colleagues (2018) created a nanoscale drug delivery system that integrated CO‐based gas therapy with PDT. They also showed how this method might be used to overcome MDR.^286^

#### 5.5.4 Chemodynamic therapy

A recently developed therapeutic approach for the treatment of cancer is CDT. By increasing oxidative stress intracellularly through Fenton or Fenton‐like reactions, CDT destroys cancerous cells or makes them susceptible to other anticancer medicines. Many metal‐containing NPs (MOFs, FeNPs), as well as iron‐incorporated NPs, have been created for CDT to this point.^287^, ^288^ Remarkably, growing research indicates that CDT can effectively enhance the therapeutic efficacy of chemotherapy and reduce MDR by inducing cellular oxidative stress. Consequently, developing combined nanomedicine and CDT agents in chemotherapy is currently a potential avenue.^289^ This is evidenced by the published finding of Guo et al. (2019), wherein doxorubicin and metal‐phenolic complex matrix was used to combat MDR.^81^ It has been shown that incorporating doxorubicin into the metal‐phenolic network blocked drug efflux transporters on chemoresistant cancer cells. This ensured the effective delivery of doxorubicin to the nucleus followed by Fenton‐induced ferroptosis. Knowledge on factors that hinder the catalytic efficiency of Fenton/Fenton‐like reactions can help to improve the CDT therapeutic agents. To decrease the occurrence of side effects, it is necessary to enhance the biocompatibility and specificity of CDT.^290^ CDT directly employs endogenous metabolism to induce oxidative stress and eradicate cancer cells. However, CDT's effectiveness is limited by ROS's limited diffusion distance and short half‐life. Many newly developed mitochondrial targeting CDT (M‐CDT) nanodrugs show great spatial specificity and anticancer effects.^291^ The development of CDT agents and chemo drugs‐integrated nanoscale drug delivery systems is thus currently a promising field of study. Chen et al. proposed a three‐pronged approach to chemotherapy, which involved combining chemotherapeutic agent with starvation therapy and CDT. This was achieved by loading cisplatin and glucose oxidase in a nanovesicle containing ferrocene.^292^

## 6 FUTURE PERSPECTIVES

**FIGURE 4:**
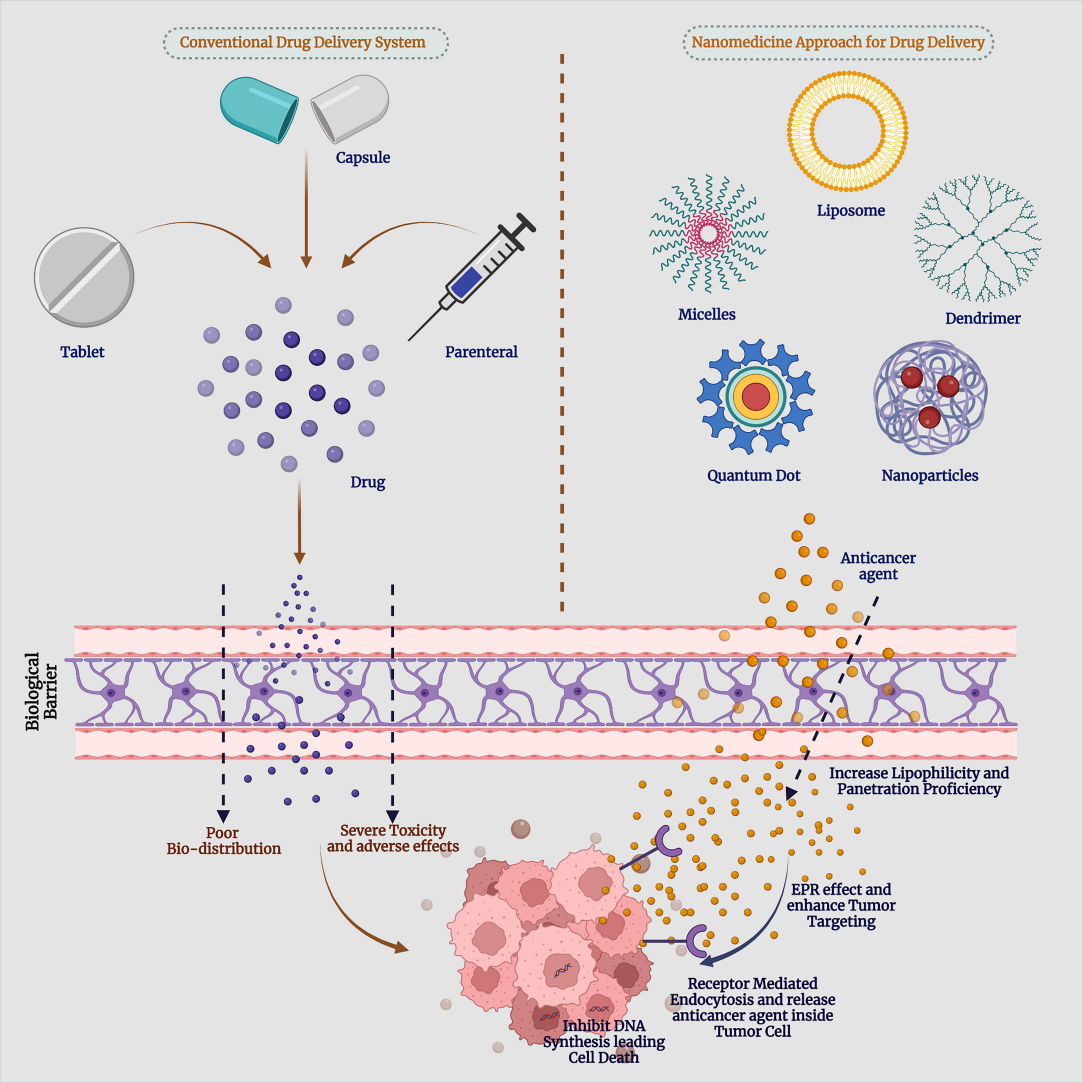
Nanoparticle‐based approaches to cancer cell targeting.

Thanks to nanotherapeutics and the development of nanocarriers that respond to specific stimuli, significant progress has been achieved in combating MDR cancers. The nano‐based approach has accomplished surmounting the obstacles of conventional drug delivery systems (Figure 4).

Owing to the significant heterogeneity of TME, some difficulties need to be addressed. The causes of MDR are also extremely intricate and might differ between people and tumor cells. Even though non‐targeted nanocarriers can accumulate by taking advantage of the EPR effect, it must be kept in mind that most research is done on in vivo animal models, which do not accurately mirror tumor progression inside a human body. Therefore, it is crucial to create actively targeted nanomedicines, which include precise drug release and delivery. Investigations into the biocompatibility and safety of nanomaterials and additional studies into MDR pathways are needed. Personalized nano therapies for cancer patients could be anticipated in the near future to combat MDR. A major challenge in the development of innovative nanomaterials to treat chemoresistant cancers is achieving high efficacy against cancer while maintaining safe systemic biocompatibility of the drug delivery system in vivo. This Review article emphasized that basic, two‐ or three‐component systems cannot be used to accomplish these goals. Particularly, a straightforward drug carrier technique does not appear to be sufficient. This is because metastatic, chemoresistant cancers are challenging to treat and have a high likelihood of recurrence. Therefore, in addition to nanomaterial systems targeting cancer cells specifically, it is necessary for such systems to permit the use of other cancer‐killing processes, such as PDT or PTT, which can enhance the therapeutic impact by further sensitizing the cells. It would be ideal if such a technology could also visualize cancer cells directly within the body. This would allow for quick, profound‐resolution tracing of treatment effectiveness and cancer response. This method enables real‐time diagnosis, enabling alternative treatments as necessary.

## 7 CONCLUSIONS

MDR is currently one of the biggest obstacles in the way of effective anticancer therapy, and only combination therapy has been proven to be clinically effective in treating MDR, despite its limited success due to the high diversity that distinguishes human cancers and the inherent characteristics of the drugs used. For instance, the in vivo impacts are complicated by their various pharmacokinetic characteristics. Combination therapy is more effective when using nanomedicines because they can target multiple molecules at once, promote the regulated release, and simultaneously encapsulate multiple molecules. To date, only seven clinical trials have been reported that use NPs to manage drug‐resistant cancer patients, despite the many advantages over traditional treatments. There are still issues to be resolved before expanding the use of NPs in medical settings. In fact, it must be taken into account that depending on the complexity of the entire system, once medications have reached their intended tumor, they may function unexpectedly. It is also crucial to note that healthy organs can sequester and retain NPs, raising questions about safety and possibly provoking resistance over time. Investigation into how nanotherapeutics behave regarding tissue accumulation and safety characterization is of utmost importance as nanomaterials might behave contrarily in biotic components and can cause toxicity because of their unusual surface chemistry. Numerous open questions need to be addressed, as well as in vitro and ex vivo models that quickly and cheaply assess the toxicity of NPs, define administration routes, and simulate absorption, distribution, metabolism, as well as excretion in living organisms. Last but not least, producing nanocarriers on a large scale with precise drug ratios is still challenging, and more advancements are needed before use in the clinical setting. To ensure appropriate measures for the clinical translation of nanomedicine, the definition of ad hoc regulations continues to be a crucial issue.

## AUTHOR CONTRIBUTIONS

**Sumel Ashique:** Conceptualization (lead); investigation (equal); supervision (equal); validation (supporting); writing – original draft (lead); writing – review and editing (supporting). **Ashish Garg:** Data curation (equal); investigation (equal); resources (equal); software (equal); writing – original draft (supporting). **Arshad Farid:** Investigation (equal); writing – original draft (equal). **Afzal Hussain:** Writing – review and editing (equal). **Prashant Kumar:** Investigation (equal); writing – original draft (equal). **Farzad Taghizadeh‐Hesary:** Supervision (equal); validation (lead); writing – review and editing (lead).

## FUNDING INFORMATION

Not applicable.

## CONFLICT OF INTEREST STATEMENT

The authors declare that they have no competing interests.

## INSTITUTIONAL REVIEW BOARD STATEMENT

Not applicable.

## INFORMED CONSENT STATEMENT

Not applicable.

## Data Availability

Not applicable.
